# Supplementary material for: Copy Number Variations Contribute to Intramuscular Fat Content Differences by Affecting the Expression of PELP1 Alternative Splices in Pigs
Source: Animals (Basel). 2022 May 27;12(11):1382. doi: 10.3390/ani12111382 (PMC9179479; doi:10.3390/ani12111382)
Supplement: Supplementary file 1 [file animals-12-01382-s001.zip › Supplementary Table S1.pdf]

Table S1. Overlapped IMF associated QTLs of significant CNVRs

CNVR	Chromosome	Start	End	Type	Overlapped QTLs
CNV11	1	43146501	43151500	Del	-
CNV35	1	174451501	174455500	Del	-
CNV49	1	236168001	236172000	Del	QTL29591
CNV385	2	17955001	17967500	Dup	-
CNV422	2	82146001	82150000	Del	-
CNV450	2	142722001	142725000	Del	-
CNV466	3	25786001	25790000	Dup	-
CNV508	4	44804501	44809500	Del	QTL144/QTL18034
CNV653	7	78876501	78952500	Dup	-
CNV657	7	79216001	79273500	Dup	-
CNV698	8	91666001	91675500	Del	-
CNV148	12	49181501	49205000	Dup	-
CNV149	12	49461001	49498500	Dup	-
CNV150	12	52194501	52220000	Dup	-
CNV160	13	25532501	25537500	Dup	-
CNV223	14	20385001	20388500	Dup	-
CNV771	X	8710001	8835000	Dup	-
CNV846	X	75184001	75198000	Dup	QTL24310/QTL24315/QTL24318
CNV901	Y	5446501	5449000	Del	-
