# Supplementary material for: Copy Number Variations Contribute to Intramuscular Fat Content Differences by Affecting the Expression of PELP1 Alternative Splices in Pigs
Source: Animals (Basel). 2022 May 27;12(11):1382. doi: 10.3390/ani12111382 (PMC9179479; doi:10.3390/ani12111382)
Supplement: Supplementary file 1 [file animals-12-01382-s001.zip › Supplementary Table S2.pdf]

Table S2. Genes involved in the PPI network of PELP1

#node	coexpression	experimentally_determined_interaction	database_annotated	combined_score	annotation
AP3B1	0	0.873	0	0.873	AP-3 complex subunit beta-1
AR	0	0.393	0.9	0.977	Androgen receptor
CDK11A	0	0.847	0	0.85	Cyclin dependent kinase 11A
CREBBP	0.061	0.379	0.9	0.955	CREB-binding protein
EP300	0.061	0.379	0.9	0.956	Histone acetyltransferase p300
ESR1	0	0.433	0.9	0.995	Estrogen receptor
HGS	0	0.379	0	0.782	Hepatocyte growth factor-regulated tyrosine kinase substrate
IPO5	0.077	0.358	0	0.439	Importin-5
LAS1L	0.114	0.986	0.9	0.999	Ribosomal biogenesis protein LAS1L
MDN1	0.064	0.387	0	0.51	Midasin
NOL9	0.044	0.986	0.9	0.998	Polynucleotide 5'-hydroxyl-kinase NOL9
NOLC1	0.046	0.403	0	0.406	Nucleolar and coiled-body phosphoprotein 1
NR3C1	0	0.321	0.9	0.945	Glucocorticoid receptor
NR4A1	0	0.414	0	0.58	Nuclear receptor subfamily 4 group A member 1
RB1	0	0.379	0	0.445	Retinoblastoma-associated protein
RPL11	0.087	0.358	0.9	0.94	60S ribosomal protein L11

RPL26	0.079	0.358	0.9	0.939	Ribosomal protein L26
RUVBL1	0.11	0.41	0.9	0.942	RuvB-like 1
SENP3	0.086	0.888	0.9	0.994	Sentrin-specific protease 3
SRC	0	0.433	0.9	0.986	Proto-oncogene tyrosine-protein kinase Src
STAT3	0	0.379	0	0.682	Signal transducer and activator of transcription 3
TEX10	0.107	0.888	0.9	0.996	Testis-expressed protein 10
WDR18	0.151	0.888	0.9	0.994	WD repeat-containing protein 18
