# Supplementary material for: Copy Number Variations Contribute to Intramuscular Fat Content Differences by Affecting the Expression of PELP1 Alternative Splices in Pigs
Source: Animals (Basel). 2022 May 27;12(11):1382. doi: 10.3390/ani12111382 (PMC9179479; doi:10.3390/ani12111382)
Supplement: Supplementary file 1 [file animals-12-01382-s001.zip › Supplementary Table S3.pdf]

Table S3. Primers for CNVs and PELP1 QPCR validation

ID	Primer sequence (5′-3′)	Length (bp)	Tm(℃)
CNV150	F: GGAACTGACCTCTGGAGCCTGA<br>R: GGAAGCCACCTGACAGCGAGAT	196	60
CNV223	F: GGGTGAGAGGGAGTCCATTTGC<br>R: TTGCTGTGGCTCTGGCGTAG	170	60
CNV148	F: TGGTCATGGTGGCTTCCTCCTT<br>R: GGCTTGCTTGCTCCTGGTTCT	153	60
CNV11	F: AACTCCCAGGCATTCCCATTGT<br>R: GCTCACAGCAACACCAGATCCT	117	60
PELP1	F: ACCTACATACACCACAGCTCACA<br>R: GATAACGAATCCGACTAGGAACCAT	103	59
PELP1-AS	F: CCACTCATCATCACTCACCTCTT<br>R: CCGAAGCCAGGAGACACAGT	117	59
GCG	F: GCAATATGGCTTTAGAATACACCTCTTA<br>R: GTCACTAATCAAGATCGTGTTCACAAC	104	60
GAPDH	F: AGGGCATCCTGGGCTACACT<br>R: TCCACCACCCTGTTGCTGTAG	166	59
