# Supplementary material for: Copy Number Variations Contribute to Intramuscular Fat Content Differences by Affecting the Expression of PELP1 Alternative Splices in Pigs
Source: Animals (Basel). 2022 May 27;12(11):1382. doi: 10.3390/ani12111382 (PMC9179479; doi:10.3390/ani12111382)
Supplement: Supplementary file 1 [file animals-12-01382-s001.zip › Supplementary Table S4.pdf]

**Table S4.** Phenotype comparison among the different CNV genotypes of 19 significant CNVs

| CNVRs | genotype | mean | stdev |
|---|---|---|---|
| CNV11 | AA | 2.91 | 0.10 |
| | AG | 2.73 | 0.12 |
| | GG | 3.48 | 0.62 |
| CNV150 | AA | 3.06 | 0.12 |
| | AC | 2.65 | 0.11 |
| | CC | 2.45 | 0.14 |
| CNV657 | AC | 2.39 | 0.14 |
| | CC | 2.92 | 0.09 |
| CNV223 | AA | 2.31 | 0.08 |
| | CC | 3.37 | 0.12 |
| CNV466 | AA | 2.69 | 0.08 |
| | AC | 3.48 | 0.23 |
| | CC | 5.17 | 1.87 |
| CNV698 | AA | 2.43 | 0.08 |
| | AG | 2.88 | 0.11 |
| | GG | 3.75 | 0.27 |
| CNV846 | AA | 2.63 | 0.08 |
| | AC | 2.80 | 0.18 |
| | CC | 4.01 | 0.31 |
| CNV149 | AA | 2.93 | 0.11 |
| | AC | 2.76 | 0.16 |
| | CC | 2.77 | 0.13 |
| CNV385 | AA | 2.72 | 0.12 |
| | AC | 2.94 | 0.12 |
| | CC | 2.90 | 0.18 |
| CNV653 | AA | 2.91 | 0.08 |
| | AC | 1.93 | 0.23 |
| | CC | 2.07 | 0.13 |
| CNV771 | AA | 2.78 | 0.08 |
| | AG | 4.10 | 1.04 |
| | GG | 4.59 | 0.56 |
| CNV49 | AA | 2.77 | 0.13 |
| | AG | 2.99 | 0.12 |
| | GG | 2.56 | 0.14 |
| CNV35 | AA | 2.89 | 0.14 |
| | AG | 2.85 | 0.11 |
| | GG | 2.83 | 0.18 |
| CNV450 | AA | 2.76 | 0.29 |
| | AG | 2.75 | 0.11 |
| | GG | 2.97 | 0.12 |
| CNV422 | AA | 2.98 | 0.22 |
| | AG | 2.95 | 0.11 |
| | GG | 2.65 | 0.11 |
| CNV148 | AA | 2.44 | 0.16 |
| | AC | 2.64 | 0.09 |

| | | | |
|---|---|---|---|
| | CC | 3.31 | 0.15 |
| | AA | 2.33 | 0.29 |
| CNV901 | AG | 2.98 | 0.13 |
| | GG | 2.75 | 0.09 |
| | AA | 3.12 | 0.12 |
| CNV160 | AC | 2.59 | 0.11 |
| | CC | 2.35 | 0.17 |
| | AA | 2.86 | 0.10 |
| CNV508 | AG | 2.90 | 0.13 |
| | GG | 2.45 | 0.22 |
